# Superior Orthonasal but Not Retronasal Olfactory Skills in Congenital Blindness

**DOI:** 10.1371/journal.pone.0122567

**Published:** 2015-03-30

**Authors:** Lea Gagnon, Abd Rahman Alaoui Ismaili, Maurice Ptito, Ron Kupers

**Affiliations:** 1 School of Optometry, University of Montreal, Montreal, Quebec, Canada; 2 Brain Research and Integrative Neuroscience Laboratory, Department of Neuroscience and Pharmacology, Panum Institute, University of Copenhagen, Copenhagen, Denmark; 3 Laboratory of Neuropsychiatry, Psychiatric Centre Copenhagen, Rigshospitalet, Copenhagen, Denmark; Technical University of Dresden Medical School, GERMANY

## Abstract

Sight is undoubtedly important for finding and appreciating food, and cooking. Blind individuals are strongly impaired in finding food, limiting the variety of flavours they are exposed to. We have shown before that compared to sighted controls, congenitally blind individuals have enhanced olfactory but reduced taste perception. In this study we tested the hypothesis that congenitally blind subjects have enhanced orthonasal but not retronasal olfactory skills. Twelve congenitally blind and 14 sighted control subjects, matched in age, gender and body mass index, were asked to identify odours using grocery-available food powders. Results showed that blind subjects were significantly faster and tended to be better at identifying odours presented orthonasally. This was not the case when odorants were presented retronasally. We also found a significant group x route interaction, showing that although both groups performed better for retronasally compared to orthonasally presented odours, this gain was less pronounced for blind subjects. Finally, our data revealed that blind subjects were more familiar with the orthonasal odorants and used the retronasal odorants less often for cooking than their sighted counterparts. These results confirm that orthonasal but not retronasal olfactory perception is enhanced in congenital blindness, a result that is concordant with the reduced food variety exposure in this group.

## Introduction

Chemicals can reach the nasal epithelium using the orthonasal or the retronasal route. The orthonasal route brings odorants from the environment to the nasal cavity via the nostrils during inspiration (or sniffing). The retronasal route, on the other hand, conveys odorants from the mouth to the nasal epithelium via the nasopharynx during exhalation. Although molecules can reach the nasal epithelium using these two routes, the associated perceptions often differ. For example, while freshly brewed coffee has a delightful perfume, its flavour may seem comparingly disappointing. On the contrary, a cheese like Époisses with the repulsive smell of sweaty shoes has a delicious flavour once inside the mouth. This is referred to as the “olfactory duality” of odorants referred to the mouth (internal body) or the external world [1].

There is strong evidence that vision can influence orthonasal olfaction [2–5], taste [6, 7] and flavour [8–13] perception. However, the impact of vision on retronasal olfaction alone remains largely unexplored. A study by Koza and collaborators (2005) showed that color increases intensity ratings when odorants are delivered orthonasally, but has the opposite effect following retronasal delivery [14]. These findings suggest that vision affects the ortho- and retronasal pathways differently, supporting the relative independence of the two routes.

Research from our and other laboratories has shown that visual deprivation from birth leads to higher odour awareness [15], better orthonasal detection, discrimination and/or identification skills [15–19], but lower taste abilities [20] when compared to a matched control group of sighted subjects. We suggested that the reduced taste abilities are related to various blindness-related obstacles when shopping, cooking and finding foods [20, 21], all of which contribute to underexpose the tongue to a variety of taste and flavour stimuli. The objective of the current study was to test the hypothesis that congenitally blind subjects have increased orthonasal together with decreased retronasal odour identification skills. As the identification of individual ingredients is necessary for preparing a dish, we further hypothesized that blind individuals would use the (retronasal) odorants less frequently than sighted when cooking.

## Material and Methods

### Participants

A total of 12 congenitally blind (4 females; [mean ± SEM] 42 ± 4 years; body mass index (BMI): 25.2 ± 1.5 kg/m2) and 14 sighted control (5 females; 40 ± 4 years; BMI: 23.6 ± 0.8 kg/m2) subjects participated in the study. Table 1 summarizes the demographic data and causes of blindness. All participants were asked to avoid eating strong foods (e.g. chili, garlic) 24h before the experiment, not to use perfume the day of the experiment and refrain from eating, drinking (except water) and chewing gum at least 1h prior to testing. This study was conducted in accordance with the Declaration of Helsinki. The research ethics committee of the Capital region of Denmark approved the study [H-2-2013-058] and all subjects gave informed and written consent prior testing.

**Table 1:** Demographic data of blind participants.

Sex	Age (y)	Education (y)	Etiology of blindness	Onset of blindness	Residual vision	Cooking frequency
F	26	16	Retinopathy of prematurity	Birth	None	Rarely
F	31	13	Retinopathy of prematurity	Birth	None	Once a day
F	45	15	Retinopathy of prematurity	Birth	Shapes (OS)	Rarely
F	64	10	Retinopathy of prematurity	Birth	None	Rarely
M	25	12	Retinopathy of prematurity	Birth	None	Rarely
M	29	13	Retinopathy of prematurity	Birth	None	Rarely
M	38	17	Optic nerve atrophy	Birth	None	Few times a week
M	39	12	Unknown	Birth	None	Few times a month
M	42	16	Retinopathy of prematurity	Birth	Light	Few times a day
M	45	15	Meningitis	1 year	Light, shapes	Few times a day
M	53	14	Retinopathy of prematurity	Birth	None	Few times a month
M	61	16	Retinopathy of prematurity	Birth	None	Few times a week

F, female; M, male; y, years; OS, left eye.

### Testing procedure

Grocery store condiments and other food items available or grinded into powder form (e.g. dried vegetables, candies, spices, etc.) were used as olfactory stimuli, following the protocol of Heilman and colleagues (2002). As the current study investigated differences between two normosmic populations, we extended the original 20 stimuli to 38 and assigned one half to orthonasal and the other half to the retronasal set (Table 2), based upon their smell and taste intensity scores that were assessed in a pilot study.

**Table 2:** Orthonasal and retronasal stimuli.

	Orthonasal		Retronasal	
	Target item	Distractor items	Target item	Distractor items
**1**	Vanilla	Cherry, Banana, Honey	Ginger	Mustard, Paprika, Curry
**2**	Onion	Chives, Salami, Smoked Ham	Lemon	Grapefruit, Sour Cherry, Redcurrant
**3**	Mushrooms	Bread, Fish, White Wine	Bread	Sauerkraut, Pizza, Garlic
**4**	Paprika	Ginger, Curry, Mustard	Milk	Vanilla, Banana, Coconut
**5**	Smoked Ham*	Fish, Bread, Chives	Strawberry	Apple, Redcurrant, Tangerine
**6**	Cloves	Anise, Caraway, Dill	Orange	Raspberry, Strawberry, Cherry
**7**	Garlic	Ham, Chives, Celery	Cocoa	Caramel, Muscat, Juniper
**8**	Nutmeg	Cinnamon, Coffee, Cocoa	Coffee	Muscat, Cinnamon, Cocoa
**9**	Curry	Mustard, Cheese, Cucumber	Cinnamon	Caramel, Cocoa, Honey
**10**	Raspberry	Peach, Pineapple, White Grapes	Peach	Raspberry, Pineapple, Grapes
**11**	Parsley	Chives, Carrots, Celery	Banana	Milk, Vanilla, Coconut
**12**	Caraway	Cloves, Anise, Dill	Apple	Strawberry, Redcurrant, Tangerine
**13**	Juniper	Caramel, Muscat, Cocoa	(Sour) Cherry	Grapefruit, Redcurrant, Lemon
**14**	Chives	Celery, Parsley, Carrots	Caramel	Cocoa, Cinnamon, Honey
**15**	Fish	Smoked Ham, Bread, Chives	Cheese	Curry, Cucumber, Mustard
**16**	Anise	Cloves, Caraway, Dill	Tangerine	Apple, Redcurrant, Strawberry
**17**	Dill	Caraway, Anise, Cloves	Pineapple	Peach, Grapes, Raspberry
**18**	Grapes	Peach, Pineapple, Raspberry	Pizza	Bread, Garlic, Sauerkraut
**19**	Coconut	Vanilla, Milk, Banana	Celery	Chives, Parsley, Carrots

Word inside parentheses was not required to earn a point for free identification.

*Subjects who identified either “smoked” or “ham” got half a point for free identification.

All testing was carried out with participants blindfolded. We first tested orthonasal identification skills by placing the plastic vial containing the food powder 5 cm below the participant’s nose. The subject was asked to take two normal breaths and identify as fast as possible the odorant (free orthonasal identification) while the experimenter was recording the response time using a stopwatch. Following the free identification, the experimenter verbally provided 4 possible choices and the participant had to select one of them (multiple-choice orthonasal identification). The participant was then asked whether he/she was familiar with the odour (yes/no) and if he/she has used it for cooking (yes/no). Total free and multiple choice identification scores were obtained by calculating the percentage of correct answers. For each subject, we also calculated the percentage of orthonasal stimuli that were familiar and used for cooking.

After a short 10-minute break, we tested retronasal identification skills. Two mL of stimulus powder was placed on the tongue using a teaspoon, while the subject had his/her nostrils occluded. After stimulus delivery, the participant was asked to close his/her mouth, unblock his/her nostrils, breathe normally and identify the odorant (free retronasal identification) while the experimenter recorded his/her response time. We calculated the percentage of multiple-choice retronasal identification as described above. The participant was again asked about odour familiarity (yes/no) and use of the odour in cooking (yes/no). Participants rinsed their mouth following each stimulus presentation. One blind subject was not exposed to bread, milk, cocoa, caramel and cheese because he was allergic to these compounds. We also calculated the percentage of retronasal stimuli that were familiar and used for cooking for each participant.

Finally, all subjects were asked to rate their general cooking frequency on a 6-point category scale (never, rarely, few times a month, few times a week, once a day, few times a day). Cooking was defined as transforming food. For example, preparing an omelet was considered as cooking but using a microwave to warm up a dish was not.

### Analysis

Results were analysed using SPSS 21.0 (SPSS Inc, Chicago, Illinois). To test for group differences in olfactory performance or subjective experience with odours, we conducted a repeated ANCOVA with route (ortho vs. retro) as within-subject factor and group (blind vs. sighted) as between-subject factor with each of the following independent variables: free and multiple-choice identification scores, response times (for free identification) as well as the proportion of stimuli familiar to the subjects and used for cooking. Age, gender, body mass index (BMI), familiarity, usage of odorants for cooking and/or cooking frequency were considered as possible covariates. Finally, to test for group difference in cooking frequency, we conducted a Mann-Whitney U-test.

Both ortho- and retronasal olfactory functions vary as a function of gender—with women being better than men [22, 23]—and slowly decline with age [22, 24]. We hypothesized that familiarity with the stimuli and/or their use for cooking would give an advantage to olfactory skills. As we had a specific hypothesis about group advantages for the identification scores, usage for cooking and cooking frequency, we used one-tailed tests. We applied two-tailed tests for the remaining dependent variables (odour familiarity and reaction times). We used a Mann-Whitney U-test in case of non-normal distribution of the data or a t-test in case of violation of the postulate of homogeneity of regression [25]. Significance level for all statistical tests was fixed at *p* < 0.05, applying a Bonferroni correction for multiple tests.

## Results

**Fig 1:**
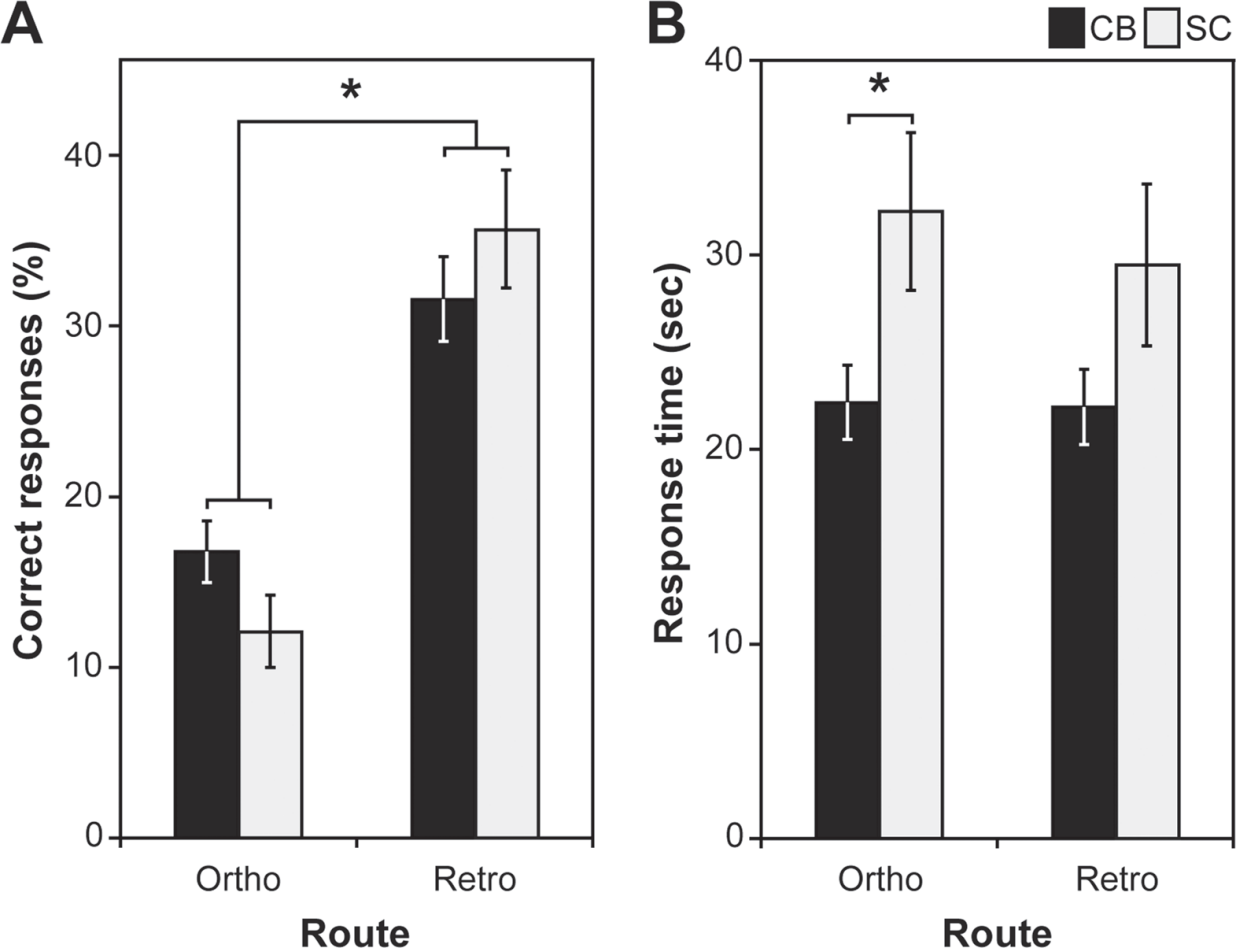
Orthonasal and retronasal odour free identification. Data are presented as mean ± standard error of the mean (SEM). A. Significant group x route interaction indicating better and worse performances respectively for orthonasal and retronasal odour free identification in congenitally blind (CB) compared to sighted controls (SC). B. Shorter response time in CB compared to sighted controls SC during the free identification task. *Significant at *p* < 0.05.

Fig 1A illustrates the mean orthonasal and retronasal free identification scores. Gender was the only significant covariate (free *p* = 0.013; multiple-choice *p* = 0.036) that had an effect on the odour identification skills. As expected, we observed a significant group x route interaction (F(23,1) = 4.696; *p* = 0.041), a trend towards a route effect (F(23,1) = 4.026; *p* = 0.057) and no group effect (F(23,1) = 0.027; *p* = 0.871). Whereas blind participants scored higher than sighted controls during orthonasal testing, they scored lower than the controls when tested retronasally. The group difference favouring the blind in orthonasal free identification almost reached significance (*p* = 0.057). For the multiple-choice identification scores, multivariate ANCOVA revealed only a trend towards a route effect (F(23,1) = 3.139; *p* = 0.090), no group effect (F(23,1) = 0.614; *p* = 0.441) and no group x route interaction (F(23,1) = 0.400; *p* = 0.533; S1 Fig). For both free and multiple-choice identification, retronasal odour identification tended to be easier than orthonasal odour identification.

Fig 1B illustrates the mean response times for both ortho- and retronasal free identification. Blind subjects were significantly faster than sighted controls in the orthonasal (t(24,1) = 2.189; *p* = 0.042) but not in the retronasal (U(24, 1) = 64.00; *p* = 0.322) task.

**Fig 2:**
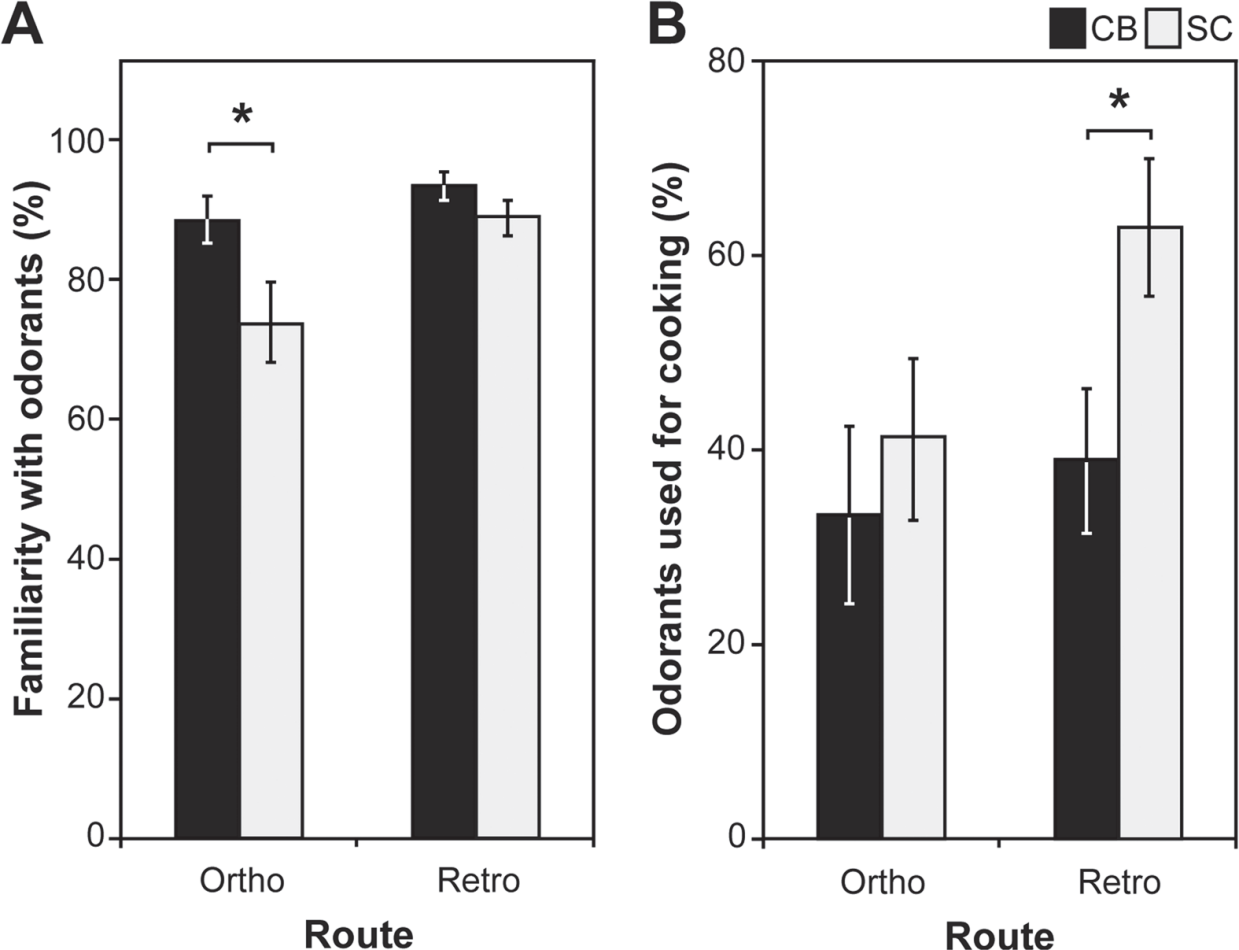
Subjective experience with odours. Data are presented as mean ± SEM. A. Congenitally blind (CB) are more familiar with the orthonasal odours and B. use less retronasal odours for cooking than sighted controls (SC). *Significant at *p* < 0.05.

Fig 2 illustrates that blind subjects were more familiar with the orthonasal odours compared to sighted controls (F(24,1) = 4.663; *p* = 0.041). There was no group difference for the retronasal stimuli (U(24, 1) = 60.50; *p* = 0.231). In line with our hypothesis, congenitally blind subjects also cooked less often with the odorants used for the retronasal testing (F(24,1) = 4.679; *p* = 0.021), whereas there was no such difference for the orthonasal odours (U(24, 1) = 73.00; *p* = 0.595).

Finally, congenitally blind individuals cooked less often than sighted controls subjects (U(24, 1) = 49.50; *p* = 0.038). Whereas more than half of the blind subjects (58%) indicated cooking a few times a month or less, half of the sighted participants (50%) reported cooking at least once a day.

## Discussion

The present data show that congenitally blind subjects are better than sighted controls at identifying odorants presented via the orthonasal but not via the retronasal route. In particular, blind subjects were faster in recognizing orthonasally presented odours and they showed a strong trend for better performance on the orthonasal free identification test. Importantly, we did find a significant route x group interaction, supporting our hypothesis that congenitally blind have enhanced orthonasal olfactory abilities but lose this behavioral advantage when smelling retronasally. Moreover, whereas blind subjects were more familiar with the orthonasal odorants, they used the retronasal odorants less for cooking, offering a possible explanation for their olfactory performances.

The olfactory system is strongly prone to both short and long-term experience-induced plasticity at cellular, synaptic and network level [26]. In the absence of vision, individuals will rely more strongly on orthonasal olfaction, as it becomes the second most important telereceptive sense after audition. Odours can be used as distal cues for wayfinding [27–29] and for social interactions with others [15, 30, 31]. Smelling the environment through the nostrils can provide representations of the actions and emotions of others [32–34]. For example, inhaling the smell of grilled meat and burning charcoal may inform that the neighbours are barbecuing. Similarly, smelling body odours enables kin and emotion recognition [35–37]. By relying more strongly on their orthonasal sense of smell, blind individuals hence come to better understand and interact with the external world [15–19]. This may also explain the increased volume of the olfactory bulb in congenitally blind individuals [19] and their stronger blood oxygenation-level dependent (BOLD) response to odorant stimuli in brain areas involved in orthonasal olfactory perception, like the amygdala, hippocampus and orbitofrontal cortex [35, 38–39]. Our results are thus in line with a variety of studies showing the superiority of congenitally blind individuals in performing orthonasal olfactory tasks.

Whereas our orthonasal sense of smell receives permanent input by a constant flow of various odours from our surrounding environment, retronasal olfactory perception relies upon the act of eating. Eating implies the search for foods, the decision of what and how much to eat and, importantly, the act of preparing food. Vision largely influences food searching and eating behaviour. Not only does the dorsal visual system enable foraging but the ventral visual stream allows rapid food identification and palatability evaluation. Although both dorsal and ventral streams remain functionally intact in the congenitally blind brain and are recruited through the remaining sensory modalities [40–42], navigational skills are impaired [43]. In modern Western urban societies, food identification prior to ingestion has become more challenging, as most palatable items are packaged in such a way that its excludes olfactory or haptic exploration that may give cues about the identity of the food product. As a result, when blind subjects shop in grocery stores, they largely depend upon a third person to locate and identify food items [21, 44]. Since blind persons’ decisions to buy foods are not influenced by visual attractiveness, they buy less spontaneously and very often limit themselves to foods that they have indicated on their pre-prepared Braille grocery shopping list. This reduces the possibility of discovering new food products, as is often the case for sighted individuals. More importantly, meal preparation is also difficult without vision. Sharp knives, hot stoves and even opened doors of high cabinets are sources of multiple injuries for the visually impaired [45]. When eating, external visual cues, like the quantity of food left in the plate [46, 47], visual characteristics of the food and dishes [48–51], facial expressions and body shapes of the people with whom we eat [52], constantly influence our intake of foods. Without these cues, blind people eat slower [46], consume more intuitively and restrain less than sighted subjects [20]. We suggest that these difficulties in food searching and eating behaviour not only have downside effects on taste perception [20] but also on retronasal olfactory abilities, as demonstrated in this study.

## Conclusion

In conclusion, our results indicate that the olfactory advantage of congenitally blind over sighted controls largely depends upon the route of stimulation. Whereas blind subjects are better at the orthonasal identification of food odours, they lose their superiority when palatable odours are smelled retronasally through the pharynx. Results on familiarity with foods and their usage for cooking were concordant with perceptual differences, supporting experience-dependent olfactory plasticity. Our results encourage further research in improving access to foods, meal preparation and gastronomy for the visually impaired. As cooking and eating are social activities that largely influence quality-of-life, this could promote independence and positively affect the well-being of people suffering from visual impairments.

## Supporting Information

S1 Fig Orthonasal and retronasal odour identification. Data are presented as mean ± SEM. Congenitally blind (CB) perform equally well than sighted control (SC) subjects at identifying odours using a multiple-choice paradigm.
(TIF)
