# Supplementary figures and images for: Superior Orthonasal but Not Retronasal Olfactory Skills in Congenital Blindness
Source: PLoS One. 2015 Mar 30;10(3):e0122567. doi: 10.1371/journal.pone.0122567 (PMC4379017; doi:10.1371/journal.pone.0122567)

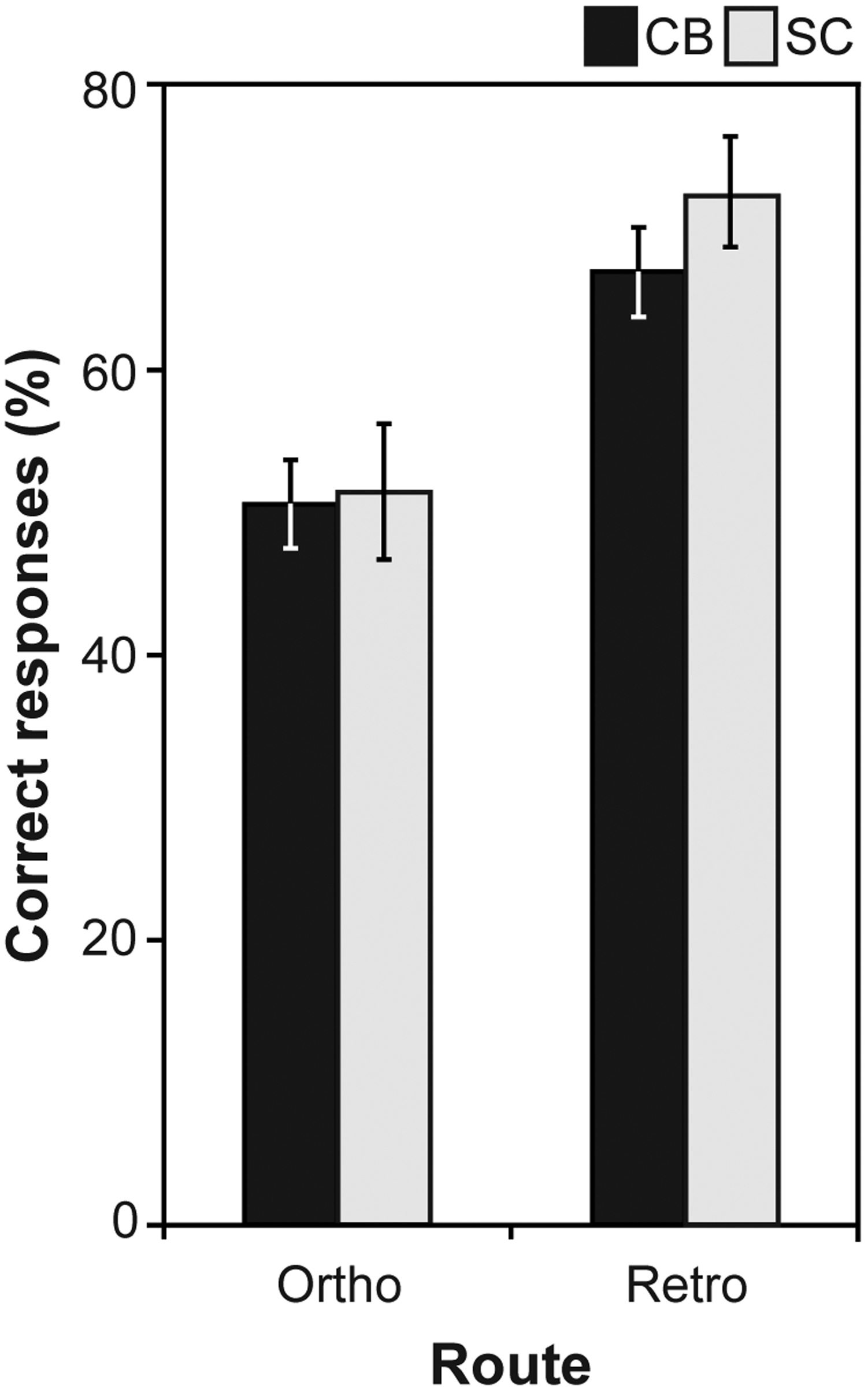

Supplement: S1 Fig — Data are presented as mean ± SEM. Congenitally blind (CB) perform equally well than sighted control (SC) subjects at identifying odours using a multiple-choice paradigm. (TIF) [file pone.0122567.s001.tif]
